# Comparative efficacy and safety of dexmedetomidine and midazolam for conscious sedation in blind nasotracheal intubation: a randomized controlled trial

**DOI:** 10.3389/fmed.2025.1689501

**Published:** 2025-11-21

**Authors:** Zhengyu Li, Man Wang, Jing Zhang, Hongjin Wu, Xue Zhang, Hong Luo, Heng Yang

**Affiliations:** Department of Anesthesiology, The Third Affiliated Hospital of Anhui Medical University, Hefei, Anhui, China

**Keywords:** blind nasotracheal intubation, conscious sedation, dexmedetomidine, midazolam, airway management, sedative comparison

## Abstract

**Objective:**

To evaluate the safety and efficacy of dexmedetomidine amnestic analgesia slow induction for blind nasotracheal intubation (BNTI) in oral and maxillofacial surgery.

**Methods:**

Sixty patients undergoing oral and maxillofacial surgery were randomly divided into the dexmedetomidine (DEX) group (1.0 μg/kg, 15 min of infusion + pethidine 1.0 mg/kg) and the midazolam (MID) group (0.02 mg/kg + pethidine 1.0 mg/kg). The intubation time was recorded, and heart rate (HR), mean arterial pressure (MAP), bispectral index (BIS) and pulse oximeter oxygen saturation (SpO_2_) were monitored at T0 (before induction), T1 (before intubation) and T2 (after intubation). Cortisol (Cor), norepinephrine (NE), epinephrine (E) and beta-endorphin (*β*-EP) levels were detected at T0, T3 (3 min after intubation) and T4 (15 min after intubation). The intraoperative fentanyl dosage, end-tidal carbon dioxide partial pressure (P_ET_CO_2_) were recorded, and postoperative numerical rating scale (NRS) score, satisfaction and adverse reaction incidence were evaluated.

**Results:**

Compared with the MID group, the intubation time in the DEX group was shorter and the P_ET_CO₂ was lower (*p* < 0.05). In the DEX group, BIS and HR were lower at T1-T2 (*p* < 0.001), and the levels of Cor, NE, E, and β-EP were lower at T3-T4 (*p* < 0.05). The dosage of sufentanil, the postoperative NRS score, and adverse reactions (nausea, vomiting, respiratory depression) were reduced in the DEX group (*p* < 0.05), and the awakening and extubation time were shorter (*p* < 0.05), but there was no difference in the incidence of sore throat and other symptoms (*p* > 0.05).

**Conclusion:**

Dexmedetomidine used for blind nasotracheal intubation for oral and maxillofacial surgery can provide comprehensive anesthesia effects, significantly reduce the stress response of tracheal intubation and the dosage of intraoperative opioids, while maintaining good hemodynamic stability.

## 1 Introduction

The primary risks associated with anesthesia in oral and maxillofacial surgery stem from the potential presence of a difficult airway, which may compromise the patient’s life safety. Preoperatively, comprehensive physical examinations and imaging studies are performed to evaluate the patient’s airway patency and ventilatory function, as well as to predict the difficulty of tracheal intubation. Appropriate techniques for anesthesia induction and intubation must be selected to ensure patient safety throughout the perioperative period. In many cases of oral and maxillofacial surgery, nasal intubation is required to accommodate the specific demands of the surgical procedure (1).

Blind nasotracheal intubation (BNTI) was developed during World War I (2, 3). Although the emergence of rapid sequence induction protocols and advanced visualization techniques has significantly reduced its clinical application (4), BNTI remains an essential skill for anesthesiologists to master. Fiberoptic bronchoscopy-assisted tracheal intubation is widely regarded as the “gold standard” for managing difficult airways (5); however, its widespread use is limited by high costs, technical complexity, and the need for specialized training (6). Therefore, in resource-limited settings such as grassroots hospitals, where access to visualization equipment is restricted, BNTI remains a valuable and feasible alternative airway management strategy for anticipated difficult airways (7, 8). Awake tracheal intubation is an established standard for managing patients with anticipated airway challenges, with well-documented safety and efficacy profiles (9). The awake analgesic slow induction technique involves the careful combination of analgesic, sedative, and hypnotic agents. This approach not only alleviates patient anxiety and fear while maintaining spontaneous respiration, but also ensures effective topical anesthesia, thereby minimizing irritation caused by the tracheal tube to the pharyngeal and tracheal mucosa. As a result, patients remain cooperative and capable of following verbal commands during the procedure. Throughout the process, patients remain in a state of comfort and safety; even in the event of intubation failure, the risk of severe airway-related complications remains low, underscoring the technique’s high safety profile.

Dexmedetomidine (DEX) is a novel and highly selective α2-adrenergic receptor agonist that primarily activates α2A adrenergic receptors in the spinal cord and brain, thereby exerting anti-sympathetic effects. It provides analgesia and sedation without respiratory depression, and its sedative state closely resembles natural non-rapid eye movement (NREM) sleep (10–12). Patients remain easily arousable and capable of cooperating with medical procedures. In addition, DEX inhibits the release of substance P and other nociceptive neuropeptides at the presynaptic level, thereby blocking the transmission of painful stimuli from the dorsal horn of the spinal cord to the central nervous system, which helps reduce the stress response associated with intubation (13, 14). Furthermore, DEX has bronchodilatory effects and reduces oral secretions (15). Despite these favorable pharmacological properties, limited evidence is currently available regarding the safety and efficacy of DEX in awake analgesic slow induction for tracheal intubation, particularly in the context of blind nasal tracheal intubation (BNTI) during oral and maxillofacial surgery. This study aims to evaluate the safety and effectiveness of DEX-based awake analgesic slow induction combined with BNTI in oral and maxillofacial surgical procedures, and to provide a scientifically sound reference strategy for managing difficult airways during the perioperative period.

## 2 Methods

### 2.1 Ethics

Following approval from the Ethics Committee of the Third Affiliated Hospital of Anhui Medical University on January 2, 2023 (Approval number: 2023-002), this study was registered on the China Clinical Trial Registry website (https://www.chictr.org.cn/index.html) on May 26, 2023 (Registration number: ChiCTR2300071838).

### 2.2 Patients

This study was conducted from June 1, 2023, to February 28, 2024. A total of 60 patients who underwent elective oral and maxillofacial surgery were enrolled. The primary diagnoses included maxillary, mandibular, zygomatic bone, and zygomatic arch fractures, among others.

Inclusion criteria were as follows: (1) patients aged 18 to 60 years; (2) patients classified as ASA I or II according to the American Society of Anesthesiologists classification; (3) patients undergoing nasal intubation due to mouth opening limitation or for intraoral surgical procedures.

Exclusion criteria were as follows: (1) presence of neurological diseases or severe cardiac or pulmonary conditions; (2) skull base fractures or known/suspected cerebrospinal fluid leakage; (3) history of sinusitis, nasal tumors, nasal or paranasal sinus deformities, or prior nasal surgery; (4) preoperative administration of sedatives or analgesics; (5) presence of coagulation disorders or significant bleeding tendency. All participants were fully informed about the purpose of the study, as well as potential benefits and risks, prior to signing the written informed consent form.

### 2.3 Randomization and blinding

All eligible patients were randomized using Excel to generate a randomization sequence and divided into the MID group and DEX group in a ratio of 1:1. Randomization was done by an anesthesiologist who was not involved in the subsequent stages of the experiment. Allocation concealment was implemented using sequentially numbered sealed envelopes. All participants were blinded to the treatment allocation after enrollment in the study, and all BNTI procedures were performed by the same group of experienced anesthesiologists who were blinded to the group information. Unblinding was done after the postoperative follow-up. Data collection and postoperative follow-up were performed by an anesthesiologist who was blinded to the group allocation (i.e., whether the patient received dexmedetomidine or midazolam).

### 2.4 Anesthesia and perioperative analgesia management

Upon entering the operating room, peripheral venous access was obtained for the patient. The left radial artery was punctured and cannulated under local anesthesia. The arterial pressure transducer was then connected. Vital signs, including heart rate (HR), mean arterial pressure (MAP), bispectral index (BIS), pulse oximeter oxygen saturation (SpO_2_), and the end-tidal carbon dioxide partial pressure (P_ET_CO_2_), were evaluated using a Philips IntelliVue MP-50 anesthesia monitor (Germany). A cotton swab, which had been soaked in a mixture containing 1% ephedrine (from the Northeast Pharmaceutical Group Shenyang No. 1 Pharmaceutical Co., Ltd., Shenyang; batch No. 220401) and 1% tetracaine (from Nanjing Xinbai Pharmaceutical Co., Ltd., Nanjing; batch No. 191106), was inserted into the well-ventilated side of the nasal meatus for a period of 5 min. This was done to constrict the nasal mucosal blood vessels and to enhance topical nasal anesthesia. At the same moment, a 7% lidocaine aerosol (Guangzhou Xiangxue Pharmaceutical Co., Ltd., Guangzhou; batch No. 202210002) was used for topical glossopharyngeal anesthesia. All patients inhaled pure oxygen at a rate of 5 L/min through a face mask.

**Figure 1:**
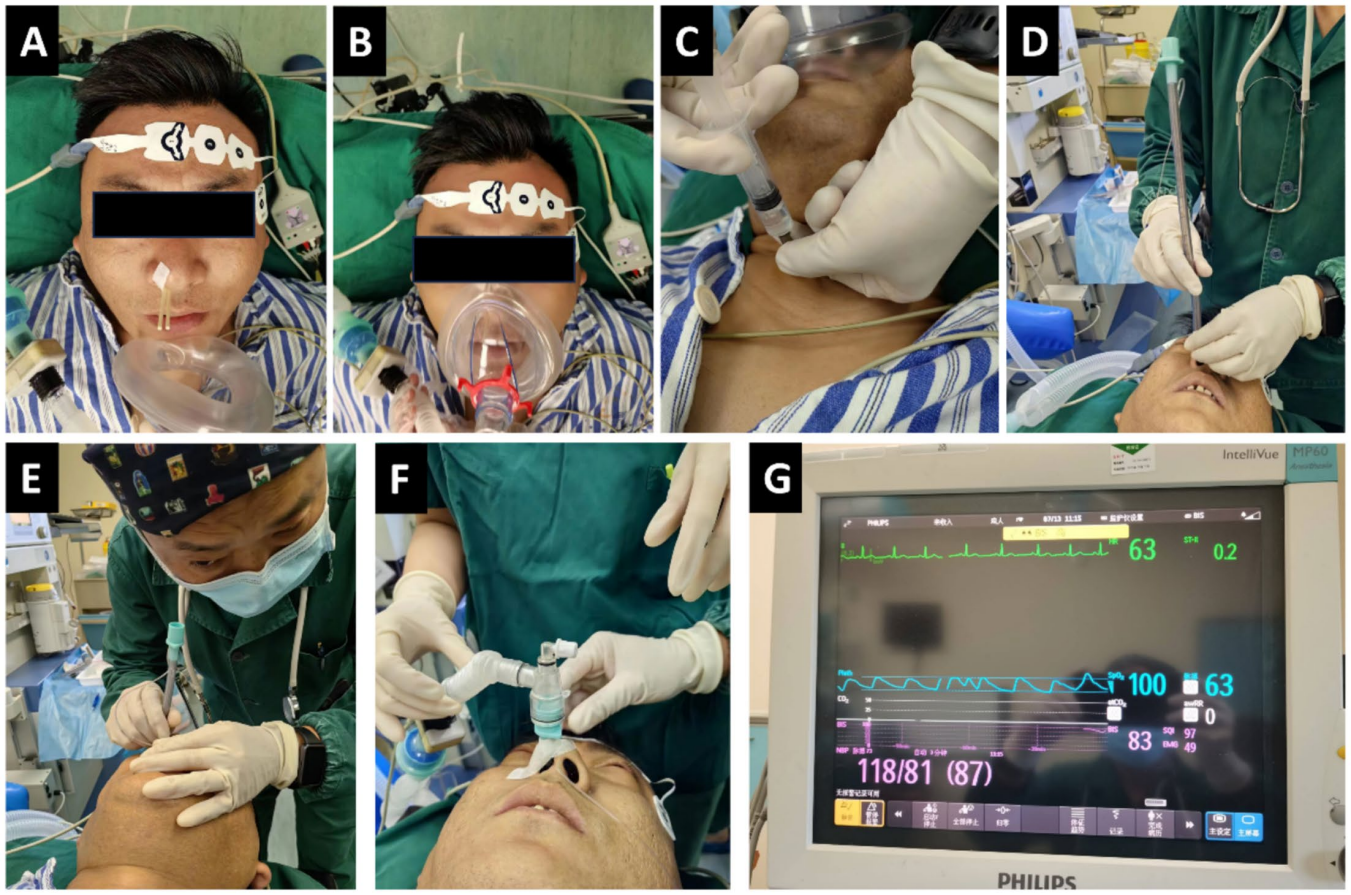
Blind nasotracheal intubation (BNTI) with slow induction anesthesia. **(A,B)** Topical nasal and glossopharyngeal anesthesia. **(C)** Cricothyroid puncture and injection. **(D,E)** BNTI. **(F,G)** Successful intubation and the patient is still conscious.

For the DEX group, dexmedetomidine (Jiangsu Hengrui Pharmaceutical Co., Ltd., Lianyungang; batch No. 10061434) was administered intravenously at a dose of 1.0 μg/kg through a continuous infusion pump for 15 min, and pethidine (Qinghai Pharmaceutical Factory Co., Ltd., Qinghai; batch No. 210401-1) was given intravenously at a dosage of 1.0 mg/kg. For the MID group, intravenous injections of midazolam (Jiangsu Enhua Pharmaceutical Co., Ltd., Xuzhou, China; batch No. MD221102) at a dosage of 0.02 mg/kg and pethidine at a dosage of 1.0 mg/kg were administered. When the BIS was in the range of 70–85, 2 mL of 1% tetracaine was injected into the trachea via cricothyroid puncture. Patients were instructed to cough in order to enhance diffusion of the drug and fully anesthetize the trachea and vocal folds. Subsequently, a reinforced endotracheal tube with an internal diameter (ID) of 6.0–7.5 mm was selected and adjusted to ensure appropriate lubrication and cuff pressure. During intubation, the patient’s head was positioned in the “sniffing” position and they were reminded to breathe deeply through the nose. The anesthesiologists held the tube in their right hand and used their left hand to adjust the position of the patient’s head, angling the tip of the tube towards the patient’s head. After the tracheal tube was passed through the nasal cavity into the pharynx, blind advancement was initiated. At this point, the operator placed their ear close to the proximal end of the tube and carefully listened to the patient’s respiratory airflow sounds. The intensity and pitch of the airflow sound vary depending on the position of the tube tip: a loud, clear airflow sound is heard when the tip is aligned with the glottis, whereas the sound significantly diminishes or disappears when the tip deviates (e.g., into the piriform fossa or against the epiglottis). Therefore, the operator subtly rotates the tube hub and makes fine anterior–posterior adjustments, continuously seeking and advancing toward the direction of the strongest and clearest airflow sound. Successful intubation is typically achieved by gently advancing the tube into the trachea during inspiration, coinciding with the point of maximal airflow sound (Figure 1).

At the time of intubation, a 10 mg dose of uradil (Xi’an Lijun Pharmaceutical Co., Ltd., Xi’an; batch No. 2203131) or 2 mg of dobutamine (Hefei Future Drug Development Co., Ltd., Hefei; batch No. 2207036) was administered to patients who experienced *a* > 20% increase or decrease in MAP from baseline values. For tachycardia or bradycardia, defined as a heart rate (HR) of more than 100 beats per minute or less than 50 beats per minute, 25 mg of esmolol (Qilu Pharmaceutical Co., Ltd., Jinan; batch No. 2L0542004) or 0.5 mg of atropine (Anhui Changjiang Pharmaceutical Co., Ltd., Wuhu; batch No. 22021306) was administered intravenously, respectively. In the case of respiratory depression, all patients underwent continuous monitoring of vital signs throughout the sedation induction period and during the blind nasotracheal intubation procedure. This included continuous electrocardiography, non-invasive blood pressure (measured at 3-min intervals), pulse oximetry, and respiratory rate monitoring. The respiratory rate was primarily recorded by clinically observing chest wall movement. Should the respiratory rate fall below 12 breaths per minute and/or the pulse oximetry (SpO₂) fall below 90%, the operator would immediately implement intervention measures, including encouraging or assisting the patient’s breathing and administering oxygen via a face mask.

Anesthesia maintenance was performed via continuous intravenous infusion of etomidate and remifentanil. During surgery, cisatracurium (0.02–0.05 mg/kg) and sufentanil (5–10 μg per administration) were intermittently administered to maintain a ventilation rate of 12–14 breaths/min, P_ET_CO_2_ levels between 35 and 45 mmHg (1 mmHg = 0.133 kPa), and BIS values within the range of 40–60. The infusion rates of anesthetic agents were adjusted intraoperatively based on the patient’s response to surgical stimuli and real-time BIS monitoring. Extubation was performed once the patient met the following criteria: regained consciousness, restored swallowing reflex, full recovery of spontaneous breathing, tidal volume (VT) >8 mL/kg, room air SpO_2_ >95%, and P_ET_CO_2_ <45 mmHg. Following extubation, patients were transferred back to the ward. Due to the increased risk of postoperative respiratory depression and airway obstruction associated with oral and maxillofacial surgery, none of the patients received postoperative patient-controlled analgesia.

### 2.5 Outcomes

The primary outcome of this study was the duration of tracheal intubation, defined as c. Secondary outcomes included: the first-attempt intubation success rate (defined as successful tracheal intubation without interruption due to SpO₂ <90%); immediate post-intubation P_ET_CO₂ levels; and the incidence of respiratory depression or epistaxis during intubation. Heart rate (HR), mean arterial pressure (MAP), bispectral index (BIS), and oxygen saturation (SpO₂) were recorded at three time points: before anesthesia induction (T₀), immediately before intubation (T₁), and immediately after intubation (T₂). Stress response indicators were assessed by collecting arterial blood samples at T₀, 3 min after intubation (T₃), and 15 min after intubation (T₄), with measurements of cortisol (Cor), norepinephrine (NE), epinephrine (E), and β-endorphin (β-EP) levels. Blood samples were sent to the Medical Laboratory of the Third Affiliated Hospital of Anhui Medical University, where serum hormone levels were analyzed using radioimmunoassay. Intraoperative sufentanil dosage was recorded. Postoperative follow-up included NRS (Numerical Rating Scale) pain scores at 2-, 6-, 12-, and 24-h post-procedure. The NRS score ranges from 0 (no pain) to 10 (severe pain), with scores of 1–3 indicating mild pain, 4–6 moderate pain, and 7–10 severe pain. Rescue analgesia with intravenous clonixin (4 mg) was administered if the NRS score was ≥4, and the number of rescue analgesia episodes within 24 h was documented. The Bruggrmann Comfort Scale (BCS) score was assessed at 24 h post-surgery, using a 0–3 scale: 0 (no discomfort), 1 (mild discomfort), 2 (moderate discomfort), and 3 (severe discomfort). Additionally, the incidence of sore throat within 24 h after surgery and the occurrence of implicit memory were recorded.

### 2.6 Sample size and statistics analysis

This study was designed as a blinded randomized controlled trial. The anesthesiologist performing the intubation and data collection was blinded to the group assignment throughout the procedure and postoperative follow-up period. With the DEX group serving as the experimental group and the MID group as the control group. The primary outcome of interest was intubation time. Based on data from a preliminary pilot study conducted at our institution involving 14 patients (seven per group), which yielded a mean difference in intubation time of 15.3 s and a pooled standard deviation of 17.2. Assuming a two-sided significance level (*α*) of 0.05 and a statistical power (1−*β*) of 0.9, and maintaining a 1:1 allocation ratio between the groups, sample size calculations were performed using R software according to the method described by Chow et al. (16). The required sample size was estimated to be 27 participants per group. To account for a potential 10% dropout or refusal rate, a total of 60 participants were ultimately enrolled, with 30 in each group.

All continuous data were first assessed for normality using the Shapiro–Wilk test. Based on the results of the normality test and the sample size, all continuous data are presented as mean ± standard deviation (SD) and were analyzed using parametric tests. All measurement data were analyzed using SPSS 23.0. Comparisons between the two groups were performed using one-way analysis of variance (ANOVA), followed by pairwise comparisons with the LSD-*t* test. For repeated measurements over time, pairwise t-tests were used to assess within-group changes across time points. The statistical significance level was set at *α* = 0.05 (two-sided). To account for multiple comparisons in repeated measures and split-plot analyses, the Bonferroni correction method was applied to adjust the significance level accordingly.

## 3 Results

### 3.1 Patient enrollment details

**Figure 2:**
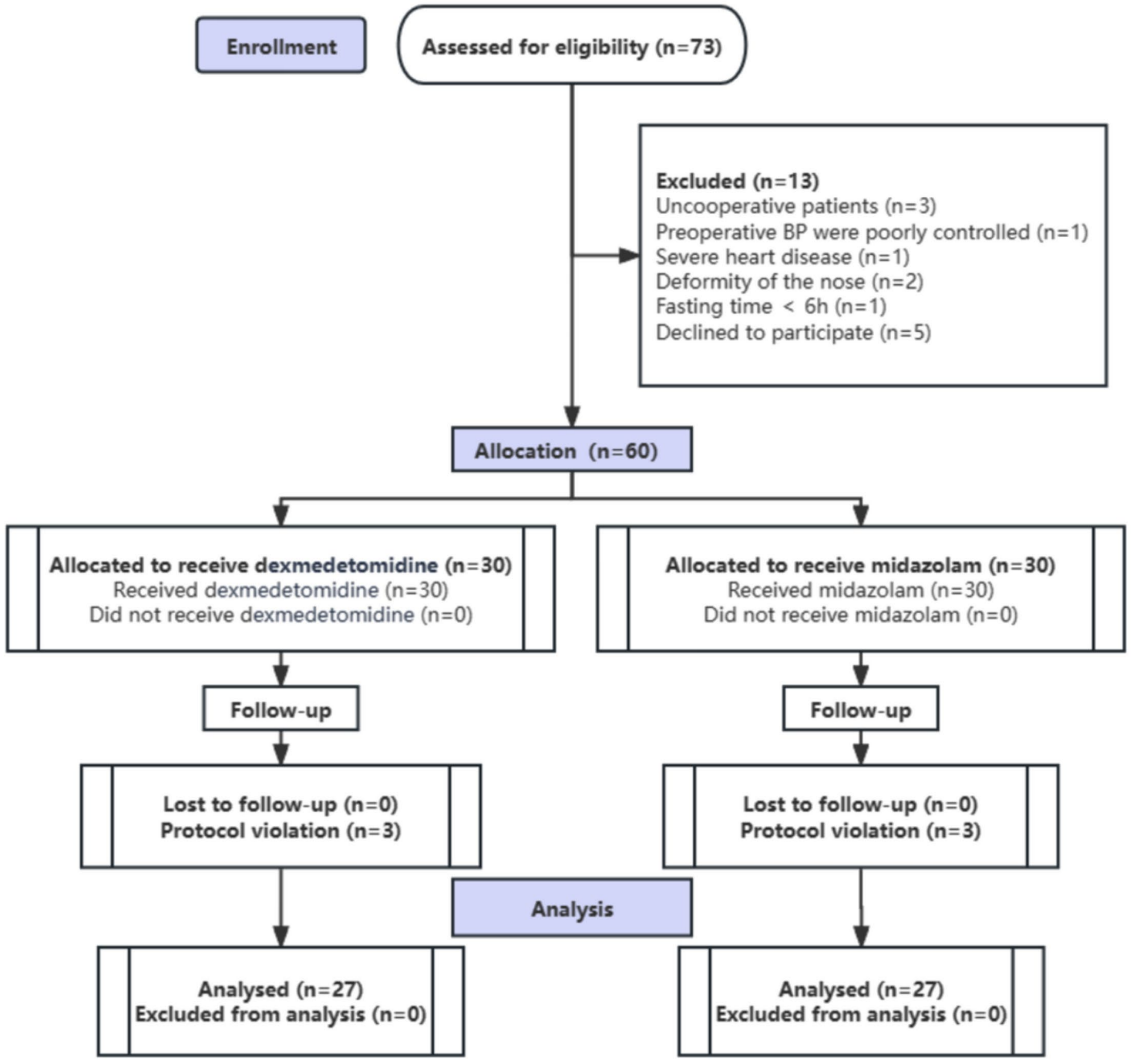
Study flow diagram of the randomized trial, including the enrollment process, assignment of interventions, and analysis (*n*, number of cases).

Among the 73 patients assessed for eligibility, 13 were excluded: eight due to failure to meet inclusion criteria and five due to refusal to provide informed consent. A total of 60 patients were enrolled in the study. Of these, three patients from the DEX group and three from the MID group withdrew from the study (withdrawal of consent) during the postoperative period prior to data collection completion. Ultimately, 54 patients were included in the final analysis (Figure 2).

### 3.2 Patient characteristics

No statistically significant differences were observed in baseline characteristics between the two patient groups, including age, gender distribution, body mass index (BMI), American Society of Anesthesiologists (ASA) classification, mento-gnathion distance, and maximal interincisal opening (MIO) (*p* > 0.05) (Table 1).

**Table 1:** Demographic characteristics of patients in the two groups.

Variable	Group DEX (*n* = 27)	Group MID (*n* = 27)	*p*
Age (years)	41.63 ± 10.65	42.44 ± 8.97	0.762
BMI (kg^.^m^−2^)	23.60 ± 2.18	23.23 ± 2.14	0.523
Gender [*n* (%)]			0.785
Male	13 (48.15)	14 (51.85)	
Female	14 (51.85)	13 (48.15)	
ASA classification [*n* (%)]			0.702
I	5 (18.52)	3 (11.11)	
II	22 (81.48)	24 (88.89)	
Thyromental distance [*n* (%)]			0.702
<6 cm	20 (74.07)	22 (81.48)	
≥6 cm	7 (25.93)	5 (18.52)	
MIO [*n* (%)]			0.340
<3 cm	22 (81.48)	19 (70.37)	
≥3 cm	5 (18.52)	8 (29.63)	

Age and BMI were compared using independent samples *t*-tests, whereas other categorical indicators were analyzed using chi-square tests. All data are presented as the mean ± SD, number (percentage). Group DEX, dexmedetomidine group; Group MID, midazolam group. ASA, American Society of Anesthesiologists; BMI, body mass index; MIO, maximal interincisal opening.

### 3.3 Comparison of intubation duration, first-attempt intubation success rate, and post-intubation P_ET_CO₂ concentration

Compared with the MID group, the DEX group showed significantly shorter intubation duration and lower post-intubation P_ET_CO₂ concentration (*p* < 0.05) (Table 2). No significant difference was observed in the first-attempt intubation success rate between the two groups (*p* > 0.05) (Table 2).

**Table 2:** Comparison of the time for blind nasotracheal intubation, end-tidal carbon dioxide partial pressure (P_ET_CO_2_) immediately after intubation and first-attempt intubation success rate in the patients between the two groups.

Outcome measure	Group DEX (*n* = 27)	Group MID (*n* = 27)	*p*
Time for intubation (s)	82.67 ± 16.41	99.41 ± 21.88	0.002
P_ET_CO_2_ immediately after intubation (mmHg)	43.70 ± 4.00	47.30 ± 4.32	0.003
The first-attempt intubation success [*n* (%)]	22 (81.48)	19 (70.37)	0.340

All data are presented as the mean ± SD, number (percentage). Group DEX, dexmedetomidine group; Group MID, midazolam group. P_ET_CO_2_, end-tidal carbon dioxide partial pressure.

### 3.4 Comparison of hemodynamic parameters, BIS values, and SpO₂ levels at each time point between the two groups of patients

Compared with baseline (T0), both groups showed significant decreases in BIS, HR, and MAP at T1, as well as reductions in BIS, MAP, and SpO₂ at T2 (SpO₂ decreased without evidence of respiratory depression), all of which were statistically significant (*p* < 0.05). Compared with the MID group, the DEX group exhibited significantly lower BIS and HR values at both T1 and T2 time points (both parameters within normal physiological ranges), with statistically significant differences (*p* < 0.001) (Table 3).

**Table 3:** Comparison of vital signs before anesthetic induction (T_0_), immediately before intubation (T_1_) and after intubation (T_2_) of patients in the two groups.

Time	Vital sign	Group DEX (*n* = 27)	Group MID (*n* = 27)	*p*
T_0_	HR (r/s)	75.19 ± 7.45	76.22 ± 7.83	0.620
	MAP (mmHg)	93.15 ± 5.51	92.44 ± 5.07	0.627
	SpO_2_	99.26 ± 0.53	99.04 ± 0.44	0.097
	BIS	97.52 ± 1.25	97.11 ± 1.31	0.248
T_1_	HR (r/s)	66.00 ± 6.48*	71.11 ± 4.47 *	0.001
	MAP (mmHg)	87.85 ± 4.53 *	87.78 ± 5.52 *	0.957
	SpO_2_	98.96 ± 0.52	98.96 ± 0.59	1.000
	BIS	76.96 ± 5.71*	82.59 ± 4.63*	<0.001
T_2_	HR (r/s)	70.33 ± 5.82*	77.07 ± 8.35	0.001
	MAP (mmHg)	84.85 ± 6.47*	83.78 ± 5.62*	0.518
	SpO_2_	94.37 ± 0.97*	93.67 ± 1.86*	0.089
	BIS	79.96 ± 6.71*	85.81 ± 4.80*	<0.001

All data are presented as the mean ± SD. Compared within the same group (*a within-group design*) at T_0_ time, *p < 0.05. Group DEX, dexmedetomidine group; Group MID, midazolam group. HR, heart rate; MAP, mean arterial pressure; SpO_2_, pulse oximeter oxygen saturation; BIS, bispectral index.

### 3.5 Comparison of stress-related biomarkers at each time point between the two patient groups

Compared with baseline (T0), the plasma levels of epinephrine (E) and β-endorphin (β-EP) in the MID group were significantly decreased at T3 and T4 time points (*p* < 0.05). Similarly, in the DEX group, the levels of cortisol (Cor), norepinephrine (NE), E, and β-EP were significantly reduced at T3 and T4 compared with T0 (*p* < 0.05). When comparing the two groups, the DEX group showed significantly lower levels of Cor, NE, E, and β-EP than the MID group at both T3 and T4 time points (*p* < 0.05) (Table 4).

**Table 4:** Comparison of the stress hormones at three (T3) and fifteen (T4) minutes after intubation of patients in the two groups.

Time	Hormone	Group DEX (*n* = 27)	Group MID (*n* = 27)	*p*
T_0_	Cor (μg/dL)	19.07 ± 6.84	18.78 ± 4.72	0.858
	NE (ng/L)	125.12 ± 22.55	134.97 ± 29.53	0.175
	E (ng/L)	37.97 ± 8.02	39.11 ± 8.21	0.609
	β-EP (mmol/L)	1.46 ± 0.30	1.48 ± 0.27	0.702
T_3_	Cor (μg/dL)	13.69 ± 3.61*	17.65 ± 7.96	0.024
	NE (ng/L)	105.75 ± 21.24*	125.52 ± 32.81	0.011
	E (ng/L)	26.81 ± 6.94*	32.85 ± 9.31*	0.009
	β-EP (mmol/L)	1.09 ± 0.27*	1.31 ± 0.33*	0.008
T_4_	Cor (μg/dL)	15.20 ± 3.35*	18.92 ± 4.98	0.002
	NE (ng/L)	90.35 ± 18.83*	121.11 ± 29.40	<0.001
	E (ng/L)	23.87 ± 6.48*	27.78 ± 7.31*	0.042
	β-EP (mmol/L)	0.99 ± 0.25*	1.28 ± 0.20*	<0.001

All data are presented as the mean ± SD. Compared within the same group (*a within-group design*) at T0 time, *p < 0.05. Group DEX, dexmedetomidine group; Group MID, midazolam group. Cor, cortisol; NE, norepinephrine; E, epinephrine, β-EP, beta-endorphin.

### 3.6 Comparison of intraoperative sufentanil consumption, numeric rating scale (NRS) scores, Bruggemann comfort scale (BCS) scores, and incidence of adverse reactions between the two patient groups

Compared with the MID group, the DEX group exhibited a significantly lower intraoperative fentanyl dosage, reduced Numeric Rating Scale (NRS) pain scores at 2 h, 6 h, 12 h, and 24 h post-surgery, decreased Bruggemann Comfort Scale (BCS) scores at 24 h post-surgery, fewer cases requiring rescue analgesia, and significantly lower incidences of nausea, vomiting, and respiratory depression (*p* < 0.05). No statistically significant differences were observed between the two groups regarding postoperative throat pain, implicit memory, post-intubation respiratory depression, or nasal bleeding (*p* > 0.05) (Table 5).

**Table 5:** Comparison of the follow-up data of patients in the two groups.

Postoperative outcome	Group DEX (*n* = 27)	Group MID (*n* = 27)	*p*
Sufentanil consumption (μg)	23.37 ± 2.08	26.74 ± 4.59	0.001
NRS_2_	2.19 ± 0.51	3.56 ± 1.13	<0.001
NRS_6_	2.39 ± 0.64	3.96 ± 0.96	<0.001
NRS_12_	2.42 ± 0.53	3.68 ± 1.05	<0.001
NRS_24_	0.93 ± 0.48	2.15 ± 0.54	<0.001
BCS_24_	1.21 ± 0.61	2.51 ± 0.52	<0.001
Number of cases requiring rescue analgesia [*n* (%)]	2 (7.41)	11 (40.74)	<0.001
Implicit memory [*n* (%)]	0 (0.00)	0 (0.00)	1.000
Sore throat [*n* (%)]	2 (7.41)	4 (14.81)	0.665
Nausea and vomiting [*n* (%)]	1 (3.70)	9 (33.33)	0.006
Respiratory depression [*n* (%)]	2 (7.41)	11 (40.74)	0.005
Epistaxis [*n* (%)]	3 (11.11)	4 (14.81)	1.000

All data are presented as the mean ± SD or *n* (%). Group DEX, dexmedetomidine group; Group MID, midazolam group. NRS, numerical rating scale; NRS_2_, NRS_24_, NRS scores were assessed at 2 h and 24 h after surgery.

### 3.7 Comparison of operation time, anesthesia duration, recovery time, Extubation time, and fluid intake and output between the two groups

There were no statistically significant differences in the operation time, anesthesia time, fluid intake, urine output, and blood loss between the two groups. Compared with the MID group, the recovery time and extubation time in the DEX group were significantly shorter, and the difference was statistically significant (*p* < 0.05) (Table 6).

**Table 6:** Comparison of operation time, anesthesia duration, recovery time, extubation time, and fluid intake and output between the two groups.

Perioperative parameter	Group DEX (*n* = 27)	Group MID (*n* = 27)	*p*
Operation time (min)	123.25 ± 21.80	121.83 ± 27.34	0.834
Anesthesia duration (min)	146.10 ± 26.45	148.52 ± 23.1	0.722
Intraoperative blood loss (ml)	75 ± 15	78 ± 20	0.536
Urine output volume (ml)	200 ± 20	200 ± 30	1.000
Total fluid infusion volume (ml)	1,000 ± 100	1,100 ± 150	0.006
Recovery time (min)	5.24 ± 0.67	10.08 ± 0.71	<0.001
Extubation time (min)	7.14 ± 0.70	12.53 ± 0.76	<0.001

All data are presented as the mean ± SD or *n* (%). Group DEX, dexmedetomidine group; Group MID, midazolam group.

## 4 Discussion

During the slow induction of tracheal intubation with amnestic analgesia and sedation, anesthesiologists administer sedative and analgesic agents to achieve an optimal pre-intubation condition. In this state, patients maintain adequate spontaneous respiration, are able to follow medical instructions, and exhibit controlled physiological responses to intubation-related stress. Simultaneously, they demonstrate effective amnesia for noxious stimuli, with no evidence of implicit memory postoperatively (17). Blind nasal tracheal intubation (BNTI) is a critical technique for managing difficult airways, particularly in patients with cervical instability, temporomandibular joint dysfunction (limited mouth opening), or congenital or acquired upper airway anomalies (5). Patients with oral and maxillofacial fractures are frequently anticipated to have difficult airways. Therefore, this study aims to evaluate the safety and efficacy of dexmedetomidine (DEX)-induced amnestic analgesia combined with slow induction BNTI in oral and maxillofacial surgical procedures.

The bispectral index (BIS) is a widely accepted and reliable parameter for monitoring the depth of anesthesia and sedation in clinical settings. It is a processed electroencephalographic (EEG) index ranging from 0 to 100, derived through advanced signal analysis (18). BIS directly reflects the effects of anesthetic agents on the cerebral cortex, thereby indicating the level of anesthesia-induced unconsciousness (19). Specifically, a BIS value above 95 corresponds to wakefulness, 65–85 indicates light to moderate sedation, 40–65 reflects an anesthetized state with suppressed arousal responses, and below 40 suggests burst suppression patterns. During the implementation of amnestic analgesia with slow induction, continuous BIS monitoring and maintenance of values between 70 and 85 help prevent excessive sedation, ensuring that patients remain cooperative and responsive to verbal commands. Furthermore, by guiding intraoperative adjustments of drug infusion rates based on real-time BIS values, anesthesiologists can achieve more precise control of anesthetic depth. This approach minimizes the risk of delayed emergence due to overly deep anesthesia, facilitates a smooth and timely recovery, and prevents intraoperative awareness associated with inadequate anesthesia (20).

Studies have demonstrated that DEX is well tolerated and maintains stable hemodynamic profiles in patients undergoing nasal tracheal intubation via fiberoptic bronchoscopy (21, 22). Comparative studies of the sedative effects of DEX and MID in both intensive care units and outpatient surgical settings have consistently shown superior performance of DEX (20). The findings of this study indicate that, compared with the MID group, patients in the DEX group exhibited greater cooperation during intubation and required significantly shorter intubation times. This study selected “nasotracheal tube placement time” (defined as the time from insertion of the tracheal tube into the nostril to confirmed correct placement within the trachea) as the primary endpoint, based on the core objective of this research: to evaluate and compare the efficiency and difficulty of the specific technical procedure of blind nasotracheal intubation under two different sedation regimens. The entire anesthesia induction process is influenced by numerous confounding factors, including the onset speed of the study drugs, individual patient responses to the medications, and pre-procedural preparation. In contrast, the “intubation time” more purely reflects the duration required to execute the key steps of this airway management technique under a predetermined sedation level, thereby more directly illustrating the impact of the sedation regimen on the conditions for intubation. Therefore, we consider it a more precise indicator for addressing the central research question. These outcomes may be attributed to the unique pharmacological properties of DEX, a sedative-hypnotic agent that activates α2 adrenergic receptors in the ventricles and stimulates dopamine neurons in the ventral tegmental area, thereby increasing dopamine concentrations in specific cortical projection regions (23). This mechanism induces a state resembling natural sleep, allowing patients to remain easily arousable (24). DEX exerts dose-dependent sedative and anxiolytic effects, with significant sedation typically observed at plasma concentrations between 0.2 and 0.3 nanograms per milliliter. This suggests the importance of precise dosing to avoid excessive sedation. This study also found that post-intubation P_ET_CO₂ levels were lower in the DEX group than in the MID group, although the average values remained within normal physiological ranges. This observation may be related to the minimal impact of DEX on respiratory function. Notably, even at plasma concentrations as high as 2.4 nanograms per milliliter, DEX has not been associated with significant respiratory depression during sedation, and patients remain readily arousable—further supporting the notion of a natural sleep-like state (25). These findings align with those of previous studies.

Xiong et al. (26) reported that preoperative oral administration of DEX effectively alleviates anxiety in surgical patients, mitigates the stress response induced by tracheal intubation under general anesthesia, and prevents hemodynamic fluctuations. DEX exhibits a characteristic biphasic hemodynamic profile. At high plasma concentrations, it activates α2-adrenergic receptors on vascular smooth muscle, resulting in peripheral vasoconstriction and subsequent hypertension, followed by reflex bradycardia mediated by carotid or aortic baroreceptors. At lower plasma concentrations, DEX induces vasodilation and suppresses sympathetic activity through presynaptic α2-adrenergic receptor activation, which reduces catecholamine release via negative feedback mechanisms (27). Notably, its efficacy in attenuating hemodynamic stress responses during intubation surpasses that of labetalol (28). Clinical evidence also indicates that administering DEX prior to anesthesia induction can reduce both blood pressure and heart rate during intubation, although it may be associated with bradycardia (29). The hemodynamic effects of DEX can be modulated by adjusting the dosage and infusion rate.

The results of this study demonstrate that, compared with baseline (T0), both groups exhibited decreased BIS, HR, and MAP at T1, and reduced BIS, MAP, and SpO₂ at T2. Although SpO₂ declined, no signs of respiratory depression were observed. Compared with the MID group, the DEX group showed significantly lower BIS and HR values at both T1 and T2. These findings suggest that the DEX group maintained more stable hemodynamics and demonstrated better tolerance to intubation. Despite the lower heart rate in the DEX group, no adverse events were recorded. The BIS values were significantly lower in the DEX group compared to the MID group at T1 (immediately before intubation). This observation confirms that dexmedetomidine provided a deeper level of sedation prior to the noxious stimulus of intubation. This profound sedation is a key mechanism underlying the attenuated stress response observed in the DEX group, as evidenced by their more stable hemodynamic profiles (e.g., heart rate and blood pressure) during intubation. The deeper sedative state, coupled with the intrinsic analgesic properties of dexmedetomidine (30), likely contributed to the reduced analgesic consumption noted in the DEX group postoperatively. Regarding the reliability of BIS monitoring under dexmedetomidine sedation, existing literature supports its validity. Studies have shown a good correlation between BIS values and the level of sedation induced by dexmedetomidine, as it primarily acts on the same molecular targets (α2-adrenoceptors) in key regions regulating sleep and arousal, such as the locus coeruleus (31, 32). While the BIS was originally developed for volatile anesthetics, it has been demonstrated to be effective in monitoring dexmedetomidine-induced sedation, reliably distinguishing between different levels of conscious sedation (32). Therefore, the lower BIS values in our DEX group robustly indicate a greater depth of sedation, which directly facilitated the favorable outcomes of reduced stress and lower analgesic requirements.

Repeated intubation attempts in patients with difficult airways may intensify intubation-related stimulation, which can trigger sympathetic activation and increased catecholamine release (33). The present study revealed that, compared with baseline (T0), the MID group exhibited decreased levels of E and β-EP at T3 and T4. In contrast, the DEX group showed significant reductions in Cor, NE, E, and β-EP at T3 and T4 compared with T0. Moreover, these biomarker levels were significantly lower in the DEX group than in the MID group at both time points. These results indicate that DEX exerts a certain sympatholytic effect and effectively attenuates the stress response associated with intubation. Administration of DEX at a dose of 0.6–1.0 μg/kg produces analgesia by activating the medullary-spinal noradrenergic pathway. However, when administered alone at a low dose (1.0 μg/kg), DEX may increase limb movement and enhance the response to intubation stimuli (34). Therefore, this study combined DEX with pethidine to enhance sedation and analgesia and reduce the anticipated intubation score. The findings of this study are consistent with previous research and align with the established pharmacological properties of DEX.

This study demonstrated that patients in the DEX group required significantly less intraoperative sufentanil compared to those in the MID group. Postoperative NSR pain scores at 2, 6, 12, and 24 h were significantly lower in the DEX group, and the BCS score at 24 h was also reduced. Additionally, the number of patients requiring rescue analgesia was markedly decreased. These findings may be attributed to the synergistic interaction between DEX and opioids, which prolongs opioid effects and reduces the required opioid dosage. It is likely that DEX exerts analgesic effects through activation of α2 receptors in both the central and spinal nervous systems (34, 35). This study demonstrated that the sufentanil dosage in the DEX group was significantly lower than that in the MID group. Previous studies have shown that patients with difficult airways are particularly susceptible to opioid-induced respiratory depression (36). Reducing opioid administration may therefore provide clinical benefits for this patient population, a finding that aligns with the outcomes of the present study. Numerous clinical studies have shown that DEX can reduce opioid requirements and postoperative complications, supporting the principles of Enhanced Recovery After Surgery (ERAS) (37). In contrast, MID lacks analgesic properties, and some studies even suggest it may lower the pain threshold (35). Furthermore, the DEX group exhibited significantly shorter awakening and extubation times compared to the MID group. The incidence of PONV and respiratory depression was markedly lower, and the overall quality of emergence was superior. No implicit memory was detected during postoperative follow-up in any patient. These results indicate that DEX-based amnestic analgesia with slow induction provides effective preemptive analgesia, reduces postoperative opioid consumption, and extends the duration of postoperative analgesia. This approach ensures a stable anesthetic induction and surgical process, thereby facilitating faster patient recovery. As an induction agent for amnestic analgesia, DEX does not compromise respiratory or cognitive recovery during emergence or extubation readiness. It also improves patient comfort scores while significantly reducing the incidence of adverse effects such as respiratory depression, nausea, vomiting, and postoperative pain.

The combination of MID and opioids exhibits synergistic respiratory depression (38, 39). MID produces sedation, anxiolysis, and anterograde amnesia by potentiating GABA_A receptor function. Pethidine is a *μ*-opioid receptor agonist that produces dose-dependent respiratory depression through direct inhibition of the brainstem respiratory center. Despite differing mechanisms of action, these two drugs exhibit synergistic or additive effects in suppressing the central nervous system, particularly the respiratory center. This synergy implies that the combined respiratory depression when administered together is substantially greater than the sum of their individual effects. The interaction pattern between DEX and opioids differs markedly from that of MID, characterized by the “opioid-sparing effect,” which may reduce the overall burden of respiratory depression (40, 41). DEX exerts sedative, anxiolytic, and analgesic effects by activating α2 receptors in brainstem regions such as the locus coeruleus. Its sedative mechanism differs from GABAergic drugs, more closely resembling physiological sleep, with minimal impact on respiration. More importantly, DEX itself possesses analgesic properties that synergize with opioids, reducing opioid dosage requirements and consequently mitigating the dose-dependent respiratory depression directly induced by opioids. Our research initiative and hypothesis were grounded precisely in this fundamental pharmacological distinction. In our experiments, we anticipated and observed more stable respiratory parameters in the DEX group. The choice of sedation regimen for awake intubation needs to be weighed against the depth of sedation, need for analgesia, respiratory and circulatory stability, and the individual patient. In addition to MID, propofol in combination with opioids can cause significant, dose-dependent respiratory depression and even apnea, with a similar risk of respiratory and circulatory depression as MID and opioid combinations, often requiring advanced airway management (42). In contrast, regimens combining DEX and opioids are more advantageous in preserving spontaneous respiration and hemodynamic stability (especially during slow infusion of a loading dose), and are particularly indicated for sedation of awake intubated patients (43). Ketamine is an NMDA receptor antagonist characterized by its unique “dissociative anesthesia” and euphoric circulation with preservation of spontaneous breathing (44). However, a major drawback is the potential for psychiatric adverse effects such as nightmares and hallucinations (45). The combination of ketamine and DEX may be a promising strategy, as DEX is effective in reducing the psychiatric side effects of ketamine, and the two complement each other in terms of analgesia and preservation of breathing (46).

This study has several limitations. This study was conducted at the Third Affiliated Hospital of Anhui Medical University. Patient characteristics, diagnostic and treatment procedures, and available resources may differ from those at other centers, potentially limiting the generalizability of the findings to other populations. Additionally, the sample size of this study (*N* = 54) is relatively small. This is because it represents the first investigation comparing the efficacy and safety of DEX versus MID for conscious sedation during blind nasotracheal intubation. The primary objective is to provide preliminary evidence and effect size estimates for future larger-scale confirmatory studies. All operators participating in this study underwent uniform and rigorous standardized training to ensure they were familiar with and proficient in the same operational procedures and evaluation criteria. Nevertheless, we acknowledge that completely eliminating operator variability is impossible. Therefore, all tracheal intubation procedures for patients were performed by the same group of anesthesiologists with over 20 years of clinical experience to ensure consistency in the benchmark for technical proficiency. An additional potential limitation of this study is the non-use of a traditional overall patient satisfaction score. Instead, we employed the Bruggemann Comfort Scale, which is specifically designed for the postoperative state. While the BCS excellently reflects physical comfort directly related to the surgery and anesthesia, it may not encompass all aspects influencing overall satisfaction, such as non-technical factors like communication with healthcare staff or the hospital environment. However, given that this study primarily focused on the physiological and comfort outcomes of the technical procedure itself, we believe the BCS provided a more targeted and objective measure for this purpose. Future studies could consider combining the BCS with a broader satisfaction scale to obtain a more comprehensive picture of the patient experience.

## 5 Conclusion

In conclusion, DEX amnestic analgesia with slow induction demonstrates significant advantages in elective oral and maxillofacial surgery, particularly for blind nasal tracheal intubation (BNTI). It is associated with shorter intubation time, greater tolerance to intubation, and a reduced stress response. The anesthesia induction is smooth, with decreased intraoperative opioid requirements, which may help reduce or prevent postoperative complications such as nausea, vomiting, and respiratory depression. Patients experience lower postoperative pain intensity and report higher satisfaction levels. The technique offers safe, comfortable, and effective anesthesia with reliable and easily mastered procedural characteristics. It is particularly well-suited for oral and maxillofacial patients to safely and stably undergo the perioperative period. For grassroots hospitals lacking advanced visualization equipment, this approach represents a valuable and practical anesthetic option.

## Data Availability

The original contributions presented in the study are included in the article/supplementary material, further inquiries can be directed to the corresponding authors.
